# Pulmonary Infection and Colonization with Nontuberculous Mycobacteria, Taiwan, 2000–2012

**DOI:** 10.3201/eid2008.131673

**Published:** 2014-08

**Authors:** Jung-Yien Chien, Chih-Cheng Lai, Wang-Huei Sheng, Chong-Jen Yu, Po-Ren Hsueh

**Affiliations:** National Taiwan University College of Medicine Graduate Institute of Clinical Medicine, Taipei, Taiwan (J.-Y. Chien);; Ministry of Health and Welfare Chest Hospital, Tainan (J.-Y. Chien);; National Taiwan University Hospital, National Taiwan University College of Medicine, Taipei (J.-Y. Chien, W.-H. Sheng, C.-.J. Yu, P.-R. Hsueh);; C.-M. Medical Center, Liouying, Taiwan (C.-C. Lai)

**Keywords:** tuberculosis and other mycobacteria, bacteria, nontuberculous mycobacteria, NTM, Taiwan, pulmonary, colonization, prevalence, respiratory infections, MTB, Mycobacterium tuberculosis, isolation, infection

## Abstract

We analyzed samples from 13,652 patients who had respiratory cultures positive for mycobacteria in Taiwan during 2000–2012 and found that 56.9% were positive for nontuberculous mycobacteria (NTM). Whereas annual prevalence of tuberculosis decreased during the study period, prevalence of NTM disease and colonization increased, particularly among older patients and male patients.

In countries to which tuberculosis (TB) is endemic, the isolation of nontuberculous mycobacteria (NTM) from clinical specimens, especially respiratory specimens, is not uncommon (*1**–**3*). In 2011, Taiwan recorded 12,634 new TB cases (55 cases/100,000 population) and 638 TB-related deaths (2.8 deaths/100,000 population); the overall incidence of TB fell 1.7% from 2010 (http://www.cdc.gov.tw/uploads/files/201308/9590e86d-875f-4d65-8435-dbcc95d9fe6d.pdf). However, in the clinical setting of patients with suspected TB, clinicians rely on local epidemiologic knowledge to evaluate whether cultures positive for mycobacteria are *Mycobacterium tuberculosis* (MTB) or NTM. Therefore, the prevalence of isolation of *M. tuberculosis* and NTM must be investigated so that the epidemiologic features of these pathogens can be better understood. Many studies have shown that there is great geographic diversity in the distribution of NTM species (*1**,**2**,**4**,**5*). However, few studies have investigated the distribution of NTM species, including colonizers and causative pathogens, with respect to patient gender and age (*6**,**7*). 

## The Study

This study was conducted at the National Taiwan University Hospital (NTUH), a 2,500-bed tertiary medical center in northern Taiwan where 8,000 clinical visits occur daily. We evaluated all patients registered in the hospital’s Mycobacterial Laboratory database with cultures positive for NTM or *M. tuberculosis* during 2000–2012. The techniques used in the preparation of different clinical specimens for cultures of mycobacteria have been described in a previous study (*2*)*.* We used the definition of pulmonary NTM infection from the 2007 American Thoracic Society/Infections Disease Society of America NTM guideline, which encompassed 3 major components: clinical signs and symptoms, radiologic findings, and microbiologic evidence (*1*). If patients had positive cultures for both NTM and *M. tuberculosis*, we defined the patients as having *M. tuberculosis* infection only. The annual prevalence rates of NTM colonization and disease were calculated as the annual number of patients with NTM colonization and disease divided by the total number of patients who visited the NTUH, including outpatients and inpatients in each indicated year.

During January 2000–December 2012, a total of 13,652 nonduplicate isolates obtained from respiratory specimens had positive test results for mycobacteria; *M. tuberculosis* was isolated from 5,878 (43.1%) patients and NTM from 7,774 (56.9%) patients. In addition, cultures of extrapulmonary specimens from 823 patients were positive for NTM. We found a significant decreasing trend in *M. tuberculosis* isolation among positive mycobacteria cultures and a significant increasing trend in NTM isolation during the study period (p<0.01 for both trends).

Among the 3,317 patients who had NTM infections (Table 1), the most prevalent species were *M. avium-intracellulare* complex (MAC) (n = 1,377, 41.5%) and *M. abscessus* (n = 710, 21.4%). Of the 4,457 patients who had with evidence of NTM colonization, the most prevalent species were MAC (n = 1,304, 29.3%) and *M. fortuitum* (n = 1,019, 22.9%) (Table 1).

**Table 1:** Annual number of patients with pulmonary mycobacterial infection caused by *Mycobacterium tuberculosis* and infection or colonization/contamination with NTM, Taiwan, 2000–2012*

Bacterial species infection or colonization	% Patients (no.)													p value
	2000, n = 456)	2001, n = 743)	2002, n = 871)	2003, n = 839)	2004, n = 1,230)	2005, n = 1,115)	2006, n = 1,116)	2007, n = 1,266)	2008, n = 1,263)	2009, n = 1,312)	2010, n = 1,138)	2011, n = 1,201)	2012, n = 1,102)	
*M. tuberculosis* infection	63.6 (290)	61.4 (456)	53.8 (469)	56.3 (472)	55.1 (678)	47.8 (533)	42.7 (476)	37.5 (475)	33.9 (428)	31.0 (407)	34.3 (390)	37.1 (446)	32.5 (358)	<0.01
NTM infection	15.8 (72)	19.5 (145)	21.1 (184)	21.1 (177)	19.0 (234)	21.2 (236)	22.6 (252)	24.6 (312)	28.2 (356)	27.1 (356)	26.4 (300)	30.7 (369)	29.4 (324)	<0.01
*M. avium–intracellulare* complex	6.8 (31)	8.3 (62)	8.4 (73)	9.5 (80)	7.3 (90)	9.1 (101)	9.3 (104)	9.3 (118)	10.6 (134)	10.2 (134)	11.2 (127)	14.2 (171)	13.8 (152)	<0.01
* M. abscessus*	2.0 (9)	3.4 (25)	4.5 (39)	3.8 (32)	2.6 (32)	4.1 (46)	5.3 (59)	5.8 (73)	6.3 (79)	5.1 (67)	5.5 (63)	7.7 (93)	8.4 (93)	<0.01
* M. fortuitum*	3.7 (17)	2.0 (15)	2.6 (23)	3.5 (29)	4.1 (50)	2.0 (22)	2.2 (25)	2.8 (35)	4.0 (51)	4.1 (54)	3.8 (43)	4.0 (48)	2.8 (31)	0.07
* M. chelonae*	1.1 (5)	1.5 (11)	1.4 (12)	2.5 (21)	2.5 (31)	2.4 (27)	2.8 (31)	3.4 (43)	3.2 (41)	3.2 (42)	2.2 (25)	1.2 (15)	0.7 (8)	0.82
* M. kansasii*	0.7 (3)	1.3 (10)	1.3 (11)	0.6 (5)	1.0 (12)	2.2 (25)	1.8 (20)	1.3 (17)	2.2 (28)	2.0 (26)	1.9 (22)	2.0 (24)	2.8 (31)	<0.01
* M. gordonae*	0.9 (4)	1.3 (10)	1.1 (10)	0.8 (7)	0.7 (8)	0.8 (9)	0.8 (9)	0.9 (11)	1.7 (21)	2.5 (33)	1.6 (18)	1.5 (18)	0.6 (7)	0.04
Other	0.7 (3)	1.6 (12)	1.8 (16)	0.4 (3)	0.9 (11)	0.5 (6)	0.4 (4)	1.2 (15)	0.2 (2)	0	0.2 (2)	0	0.2 (2)	<0.01
*M. terrae* complex	0	0.1 (1)	0.7 (6)	0	0.1 (1)	0.2 (2)	0.1 (1)	0.3 (4)	0	0	0.2 (2)	0	0	–
* M. scrofulaceum*	0.4 (2)	0.3 (2)	0.2 (2)	0	0.2 (3)	0.1 (1)	0.1 (1)	0.2 (2)	0	0	0	0	0.2 (2)	–
* M. phlei*	0	0.5 (4)	0.1 (1)	0.4 (3)	0.3 (4)	0.1 (1)	0.1 (1)	0	0	0	0	0	0	–
* M. smegmatis*	0.2 (1)	0	0	0	0.1 (1)	0.1 (1)	0	0.5 (6)	0.1 (1)	0	0	0	0	–
* M. xenopi*	0	0.1 (1)	0.8 (7)	0	0.1 (1)	0.1 (1)	0	0	0	0	0	0	0	–
* M. flavescens*	0	0.5 (4)	0	0	0	0	0	0	0	0	0	0	0	–
* M. celatum*	0	0	0	0	0	0	0	0.1 (1)	0.1 (1)	0	0	0	0	–
* M. asiaticum*	0	0	0	0	0.1 (1)	0	0	0	0	0	0	0	0	–
* M. mageritense*	0	0	0	0	0	0	0	0.1 (1)	0	0	0	0	0	–
* M. szulgai*	0	0	0	0	0	0	0.1 (1)	0	0	0	0	0	0	–
* M. vaccae*	0	0	0	0	0	0	0	0.1 (1)	0	0	0	0	0	–
NTM colonization/contamination	20.6 (94)	19.1 (142)	25.0 (218)	22.6 (190)	25.9 (318)	31.0 (346)	34.8 (388)	37.8 (479)	37.9 (479)	41.8 (549)	39.4 (448)	32.1 (386)	38.1 (420)	<0.01
*M. avium–intracellulare* complex	3.9 (18)	6.1 (45)	6.2 (54)	9.4 (79)	5.9 (72)	13.8 (154)	10.9 (122)	10.1 (128)	10.5 (132)	9.9 (130)	9.1 (103)	9.9 (119)	13.4 (148)	<0.01
* M. fortuitum*	4.4 (20)	2.7 (20)	5.2 (45)	2.6 (22)	6.4 (79)	5.6 (62)	7.6 (85)	8.5 (108)	11.2 (142)	10.1 (132)	9.8 (111)	7.1 (85)	9.8 (108)	<0.01
* M. gordonae*	2.6 (12)	3.2 (24)	3.9 (34)	2.9 (24)	3.1 (38)	1.7 (19)	3.8 (42)	5.1 (64)	5.5 (70)	8.2 (108)	10.3 (117)	6.9 (83)	6.4 (70)	<0.01
* M. abscessus*	5.3 (24)	3.1 (23)	3.3 (29)	3.1 (26)	4.1 (50)	4.7 (52)	4.2 (47)	6.2 (78)	5.1 (65)	7.1 (93)	4.8 (55)	3.8 (46)	4.5 (50)	0.01
* M. kansasii*	1.1 (5)	0.1 (1)	1.6 (14)	1.8 (15)	1.0 (12)	1.6 (18)	4.0 (45)	2.0 (25)	2.4 (30)	2.7 (35)	2.6 (30)	2.5 (30)	2.9 (32)	<0.01
* M. chelonae*	0.4 (2)	1.2 (9)	1.3 (11)	0.6 (5)	2.6 (32)	2.5 (28)	2.1 (23)	2.5 (32)	2.6 (33)	2.7 (36)	2.3 (26)	1.7 (20)	1.1 (12)	0.08
Other	2.9 (13)	2.7 (20)	3.6 (31)	2.3 (19)	2.8 (35)	1.2 (13)	2.2 (24)	3.5 (44)	0.6 (7)	1.1 (15)	0.5 (6)	0.2 (3)	0	<0.01
*M. terrae* complex	0.2 (1)	0.9 (7)	0.9 (8)	0.6 (5)	0.8 (10)	0.5 (6)	0.4 (4)	0.9 (11)	0.4 (5)	0.9 (12)	0.5 (6)	0	0	–
* M. scrofulaceum*	0.9 (4)	0.8 (6)	0.2 (2)	0.6 (5)	0.4 (5)	0	0.7 (8)	0.3 (4)	0.1 (1)	0.1 (1)	0	0.1 (1)	0	–
* M. smegmatis*	0.2 (1)	0.3 (2)	0.2 (2)	0.1 (1)	0.3 (4)	0.1 (1)	0.4 (4)	1.3 (16)	0	0	0	0	0	–
* M. xenopi*	0.2 (1)	0	1.5 (13)	0.5 (4)	0.3 (4)	0	0.3 (3)	0.1 (1)	0	0	0	0	0	–
* M. flavescens*	0.4 (2)	0	0.5 (4)	0.1 (1)	0.2 (2)	0.3 (3)	0.1 (1)	0.3 (4)	0	0.2 (2)	0	0.1 (1)	0	–
* M. phlei*	0	0.4 (3)	0.1 (1)	0.2 (2)	0.5 (6)	0.3 (3)	0.1 (1)	0.3 (4)	0	0	0	0	0	–
* M. szulgai*	0.2 (1)	0	0	0	0.2 (3)	0	0.1 (1)	0.1 (1)	0.1 (1)	0	0	0.1 (1)	0	–
* M. vaccae*	0.7 (3)	0.3 (2)	0	0	0.1 (1)	0	0.2 (2)	0	0	0	0	0	0	–
* M. simiae*	0	0	0	0.1 (1)	0	0	0	0.2 (2)	0	0	0	0	0	–
* M. marinum*	0	0	0.1 (1)	0	0	0	0	0	0	0	0	0	0	–
* M. neoaurum*	0	0	0	0	0	0	0	0.1 (1)	0	0	0	0	0	–

*NTM, nontuberculous mycobacteria; –, not applicable (insufficient data).

**Figure 1:**
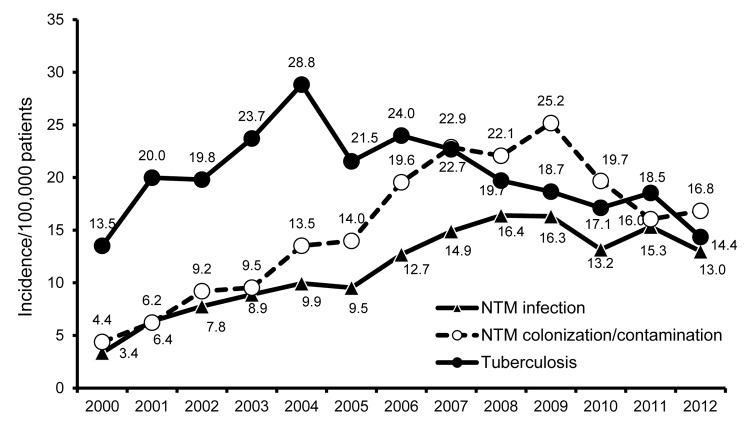
Annual incidence of tuberculosis, pulmonary nontuberculous mycobacteria (NTM) infection, and NTM colonization among patients registered in the National Taiwan University Hospital Mycobacterial Laboratory database with cultures positive for *Mycobacterium tuberculosis* or NTM, 2000–2012.

**Figure 2:**
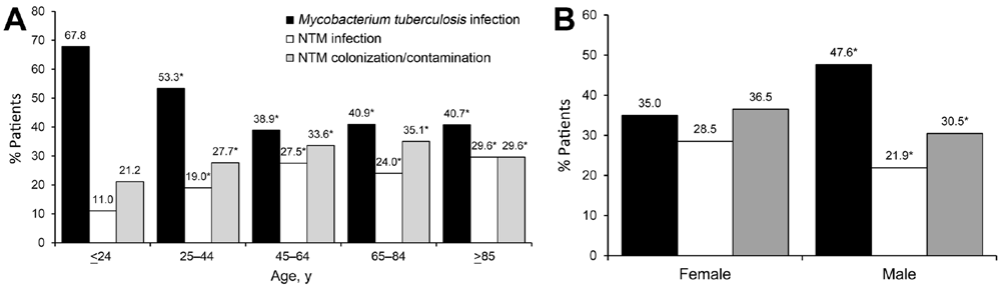
Rates of *Mycobacterium tuberculosis* infection, nontuberculous mycobacteria (NTM) infection, and NTM colonization/contamination, by age (A) and sex (B), among patients registered in the National Taiwan University Hospital Mycobacterial Laboratory database with cultures positive for *Mycobacterium tuberculosis* or NTM, 2000–2012. *p<0.01 compared with first group.

In contrast to the decreasing trend in prevalence of TB during the study period, the annual incidence of pulmonary NTM disease and colonization increased significantly over time (p<0.01) (Figure 1). In addition, the clinical significance of mycobacterial isolates differed according to patient sex and age (Figure 2); the rate of NTM isolation increased with age, whereas the rate for *M. tuberculosis* isolation decreased with age (Figure 2, panel A; p<0.01). Most cases of NTM disease and colonization were found in patients 65–84 years of age (Technical Appendix Figure), and the risk of developing NTM disease significantly increased with age (p<0.01). We also found that the male:female ratio was significantly lower among patients >45 years of age who had NTM infection or colonization than among patients in the same age group who had *M. tuberculosis* infection (p<0.01; Technical Appendix Table).

Infections caused by MAC, *M. abscessus,* and *M. chelonae* were more common in female than in male patients. In contrast, diseases caused by *M. fortuitum* and *M. kansasii* were more common in male patients than in female patients. We also found that the prevalence of infection caused by *M. tuberculosis*, MAC, *M. abscessus* (p<0.01)*, M. fortuitum* (p<0.01)*, M. chelonae* (p = 0.04)*, M. kansasii* (p = 0.04)*,* and *M. gordonae* (p<0.01) in each age group differed significantly and that the prevalence of colonization by MAC (p<0.01), *M. fortuitum* (p = 0.01)*, M. gordonae* (p<0.01)*,* and *M. kansasii* (p<0.01)in each age group differed significantly (Table 2).

**Table 2:** Bacterial species implicated in pulmonary mycobacterial infections and colonization/contamination, by patient sex and age, Taiwan, 2000–2012

Bacterial species	Sex, % (no.) patients		p value	Age, y, % (no.) patients					p value
	Female, n = 4,931	Male, n = 8,721		<24, n = 572	25–44, n = 1,792	45–64, n = 4,062	65–84, n = 6,156	>85, n = 1,070	
*Mycobacterium tuberculosis* infection	35.0 (1,725)	47.6 (4,153)	<0.01	67.8 (388)	53.3 (956)	38.9 (1,579)	40.9 (2,519)	40.7 (436)	<0.01
NTM probable infection									
*M. avium–intracellulare* complex	13.1 (645)	8.4 (732)	<0.01	3.7 (21)	6.3 (113)	11.1 (449)	10.3 (637)	14.7 (157)	<0.01
* M. abscessus*	6.8 (334)	4.3 (376)	<0.01	2.3 (13)	5.7 (102)	5.9 (241)	4.5 (275)	7.4 (79)	<0.01
* M. fortuitum*	2.7 (132)	3.6 (311)	<0.01	1.2 (7)	1.8 (33)	3.8 (155)	3.5 (218)	2.8 (30)	<0.01
* M. chelonae*	3.0 (148)	1.9 (164)	<0.01	2.6 (15)	1.9 (34)	2.9 (116)	2.0 (122)	2.3 (25)	0.04
* M. kansasii*	1.2 (61)	2.0 (173)	<0.01	0.7 (4)	2.1 (38)	1.9 (77)	1.7 (105)	0.9 (10)	0.04
* M. gordonae*	1.1 (55)	1.3 (110)	0.5	0.5 (3)	0.4 (8)	1.3 (52)	1.5 (91)	1.0 (11)	<0.01
Other	0.6 (31)	0.5 (45)	0.4	0	0.7 (12)	0.7 (28)	0.5 (31)	0.5 (5)	0.22
*M. terrae* complex	0.2 (8)	0.1 (9)	–	0	0.2 (4)	0.1 (4)	0.1 (8)	0.1 (1)	–
* M. scrofulaceum*	0.2 (8)	0.1 (7)	–	0	0	0.2 (9)	0.1 (6)	0	–
* M. phlei*	0.1 (5)	0.1 (9)	–	0	0.3 (5)	0.1 (3)	0.1 (5)	0.1 (1)	–
* M. smegmatis*	0	0.1 (10)	–	0	0.1 (1)	0.0 (2)	0.1 (5)	0.2 (2)	–
* M. xenopi*	0.1 (4)	0.1 (6)	–	0	0.1 (1)	0.0 (2)	0.1 (6)	0.1 (1)	–
* M. flavescens*	0.1 (4)	0	–	0	0.1 (1)	0.1 (3)	0	0	–
* M. celatum*	0	0.0 (2)	–	0	0	0.0 (2)	0	0	–
* M. asiaticum*	0	0.0 (1)	–	0	0	0	0.0 (1)	0	–
* M. mageritense*	0	0.0 (1)	–	0	0	0.0 (1)	0	0	–
* M. szulgai*	0.0 (1)	0	–	0	0	0.0 (1)	0	0	–
* M. vaccae*	0.0 (1)	0	–	0	0	0.0 (1)	0	0	–
NTM colonization/contamination									
*M. avium–intracellulare* complex	10.6 (525)	8.9 (779)	<0.01	4.4 (25)	6.7 (120)	9.6 (388)	10.7 (659)	10.5 (112)	<0.01
* M. fortuitum*	7.9 (389)	7.2 (630)	0.17	5.8 (33)	6.4 (114)	7.6 (310)	8.1 (499)	5.9 (63)	0.01
* M. gordonae*	6.0 (296)	4.7 (409)	<0.01	2.6 (15)	4.1 (73)	5.9 (241)	5.6 (343)	3.1 (33)	<0.01
* M. abscessus*	5.5 (273)	4.2 (365)	<0.01	4.5 (26)	4.1 (74)	5.0 (205)	4.6 (284)	4.6 (49)	0.64
* M. kansasii*	2.0 (101)	2.2 (191)	0.63	0.5 (3)	2.1 (38)	1.8 (72)	2.5 (153)	2.4 (26)	<0.01
* M. chelonae*	2.4 (120)	1.7 (149)	<0.01	2.4 (14)	2.3 (42)	1.8 (72)	2.0 (126)	1.4 (15)	0.31
Other	1.9 (96)	1.5 (134)	0.07	0.9 (5)	2.0 (35)	1.9 (77)	1.5 (94)	1.8 (19)	0.27
*M. terrae* complex	0.6 (30)	0.5 (45)	–	0.3 (2)	0.7 (13)	0.6 (25)	0.5 (30)	0.5 (5)	–
* M. scrofulaceum*	0.3 (15)	0.3 (22)	–	0	0.3 (6)	0.3 (11)	0.3 (16)	0.4 (4)	–
* M. smegmatis*	0.3 (14)	0.2 (17)	–	0	0.3 (5)	0.2 (9)	0.2 (13)	0.4 (4)	–
* M. xenopi*	0.2 (9)	0.2 (17)	–	0	0.3 (5)	0.3 (11)	0.1 (8)	0.2 (2)	–
* M. flavescens*	0.2 (11)	0.1 (9)	–	0.5 (3)	0.1 (1)	0.1 (4)	0.2 (10)	0.2 (2)	–
* M. phlei*	0.1 (6)	0.2 (14)	–	0	0.1 (2)	0.2 (9)	0.1 (9)	0	–
* M. szulgai*	0.1 (5)	0.0 (3)	–	0	0	0.1 (4)	0.0 (2)	0.2 (2)	–
* M. vaccae*	0.1 (6)	0.0 (2)	–	0	0.1 (1)	0.1 (3)	0.1 (4)	0	–
* M. simiae*	0	0.0 (3)	–	0	0.1 (1)	0	0.0 (2)	0	–
* M. marinum*	0	0.0 (1)	–	0	0	0.0 (1)	0	0	–
* M. neoaurum*	0	0.0 (1)	–	0	0.1 (1)	0	0	0	–

*NTM, nontuberculous mycobacteria ; –, not applicable (insufficient data).

## Conclusions

Our cross-sectional analysis of data from mycobacterial isolates collected over 13 years in northern Taiwan resulted in several notable findings. First, the prevalence of NTM pulmonary infection and colonization significantly increased during the period, whereas prevalence of *M. tuberculosis* infection significantly decreased. This phenomenon has been reported previously in Taiwan (*8*) and in other countries (*9*,*10*). We also found that the annual prevalence of NTM pulmonary infections and colonization significantly increased (p<0.01). The increasing trend in NTM infections has also been noted in South Korea, Canada, Denmark, Australia, the United States, and the Netherlands (*9**–**15*). These findings suggest that, although the rates of NTM pulmonary infection have gradually increased in Taiwan, more than half of NTM isolates caused colonization only.

Second, we found the frequency of isolation of *M. tuberculosis* and NTM from patients >85 years of age was 40.7% and 59.3%, respectively. Furthermore, half of NTM infections (54.1%) and colonizations (55.5%) occurred in patients >65 years of age. Our findings are consistent with those in a recent study conducted in South Korea (*7*) and suggest that most cases of NTM infection occur in patients of advanced age and that its associated clinical significance may be as important as TB among elderly patients.

Third, we found that gender may be associated with the acquisition of diseases caused by mycobacterial species. We found that *M. tuberculosis* infection, NTM pulmonary infection, and NTM colonization were more common among men than among women (70.1% vs. 29.3% for TB, 57.6% vs. 42.4% for NTM infections, and 59.6% vs. 40.4% for NTM colonization). In addition, the male:female ratio among patients >45 years of age was significantly higher among patients with TB than among patients with NTM infections or colonization. This finding might indicate that older women are more resistant to TB than to NTM infection/colonization.

This study has several limitations. First, because the study was conducted in a single medical center in Taiwan, we cannot safely generalize our findings to other parts of Taiwan. However, this hospital is the only medical center that can fully identify the species of NTM in Taiwan, which suggests that our results still provide useful information about the current status in this country. Second, we did not evaluate the effects of co-existing conditions such as HIV infection. Third, the American Thoracic Society/Infections Disease Society of America diagnostic criteria for NTM infections were determined on the basis of experience with MAC, *M. kansasii*, and *M. abscessus* but not for other NTM species (*1*)*.* Therefore, we might have overestimated the incidence of lung infections caused by other NTM species (e.g., *M. gordonae* and *M. fortuitum*). These species need further investigation. Fourth, in this retrospective analysis, we did not try to identify factors associated with the changing epidemiology of these diseases.

In summary, in Taiwan, the rate of NTM isolation among cultures positive for mycobacteria increased significantly during the period 2000–2012, whereas the rate of *M. tuberculosis* isolation decreased. Moreover, the prevalence of NTM pulmonary infection and colonization rapidly increased with time. This phenomenon was more evident among patients of advanced age and among male patients.

Technical Appendix Sex ratio by clinical significance and age group among patients with tuberculosis and colonization/contamination by nontuberculous mycobacteria and distribution of nontuberculous mycobacteria species causing infections and colonization in different age groups, Taiwan, 2000–2012.
