# Supplementary material for: Pulmonary Infection and Colonization with Nontuberculous Mycobacteria, Taiwan, 2000–2012
Source: Emerg Infect Dis. 2014 Aug;20(8):1382–5. doi: 10.3201/eid2008.131673 (PMC4111185; doi:10.3201/eid2008.131673)
Supplement: Technical Appendix — Sex ratio by clinical significance and age group among patients with tuberculosis and colonization/contamination by nontuberculous mycobacteria and distribution of nontuberculous mycobacteria species causing infections and colonization in different age groups, Taiwan, 2000–2012. [file 13-1673-Techapp-s1.pdf]

Article DOI: http://dx.doi.org/10.3201/eid2008.131673

Pulmonary Infection and Colonization with Nontuberculous Mycobacteria, Taiwan, 2000–2012

Technical Appendix

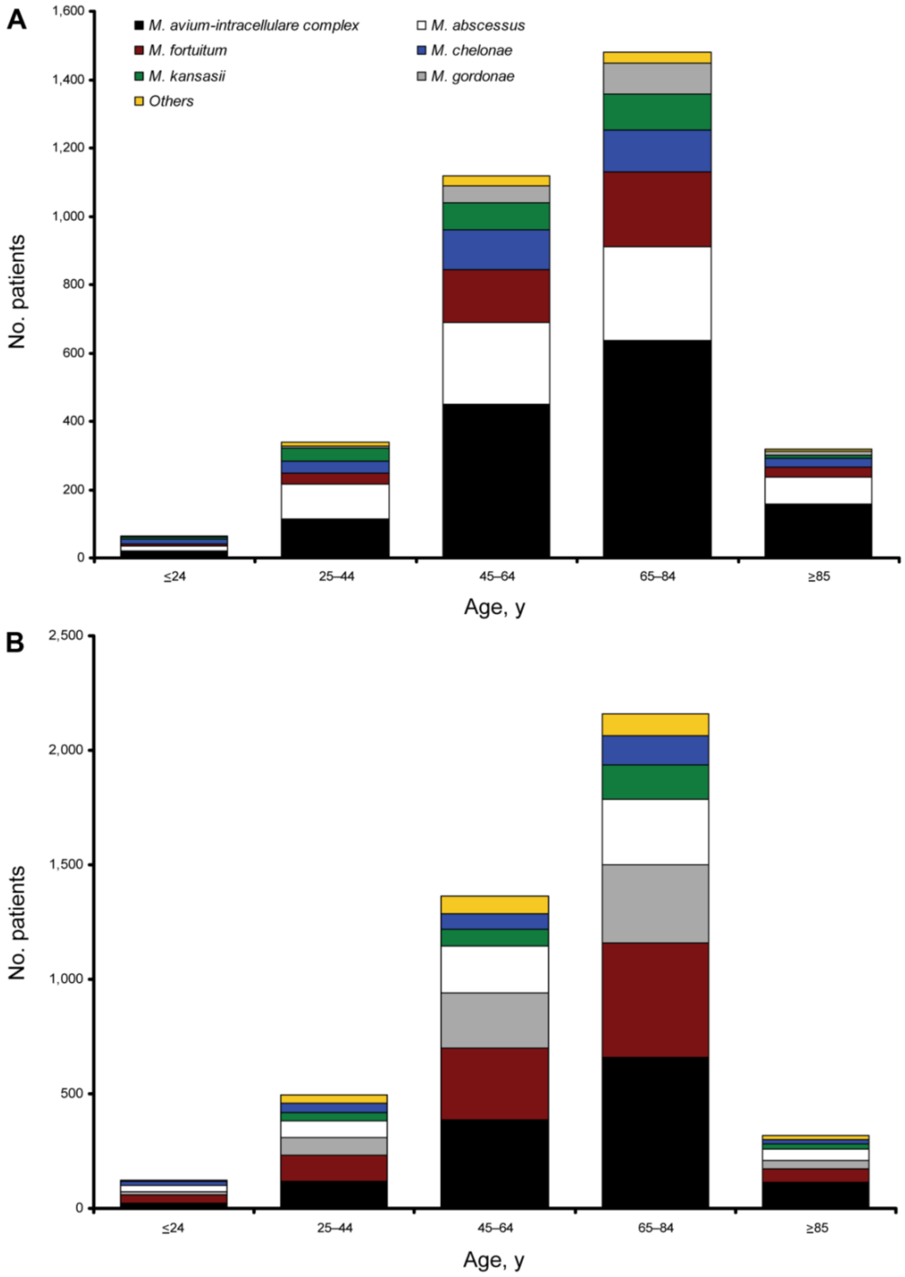

Technical Appendix Figure. Distribution of nontuberculous mycobacteria species causing infections (A) and colonization (B) in different age groups, Taiwan, 2000–2012.

Technical Appendix Table. Gender ratio according to clinical significance and age groups among patients with tuberculosis and colonization/contamination due to nontuberculous mycobacteria, Taiwan, 2000–2012*

	Male/female ratio (no. of male patients/no. of female patients)			
Age, y	Tuberculosis	NTM infection	NTM colonization/contamination	p value
≤24	1.05 (199/189)	1.86 (41/22)	1.57 (74/47)	0.04
25–44	1.16 (513/443)	1.27 (190/150)	1.11 (261/235)	0.65
45–64	2.72 (1,155/424)	0.95 (544/574)	1.16 (733/632)	<0.01
65–84	3.58 (1,969/550)	1.74 (939/540)	1.78 (1,382/776)	<0.01
≥85	2.66 (317/119)	1.64 (197/120)	1.88 (207/110)	<0.01

*NTM, nontuberculous mycobacteria.
